# Supplementary material for: Pollen extracts and constituent sugars increase growth of a trypanosomatid parasite of bumble bees
Source: PeerJ. 2017 May 9;5:e3297. doi: 10.7717/peerj.3297 (PMC5426351; doi:10.7717/peerj.3297)
Supplement: Supplemental Information 2 — Supplementary Figure 1. Growth curves for the individual pollen experiment. Supplementary Figure 2. Growth curves for the mixed-pollen experiment Supplementary figure 3. Growth curves for the chemical additions experiment. Supplementary figure 4. Effects of caffeic acid (aqueous) on three strains of Crithidia bombi. [file peerj-05-3297-s002.docx]

Supplementary Figures for

Palmer-Young EC

Pollen extracts increase growth of a trypanosome parasite of bumble bees

Supplementary Figures S1-S3


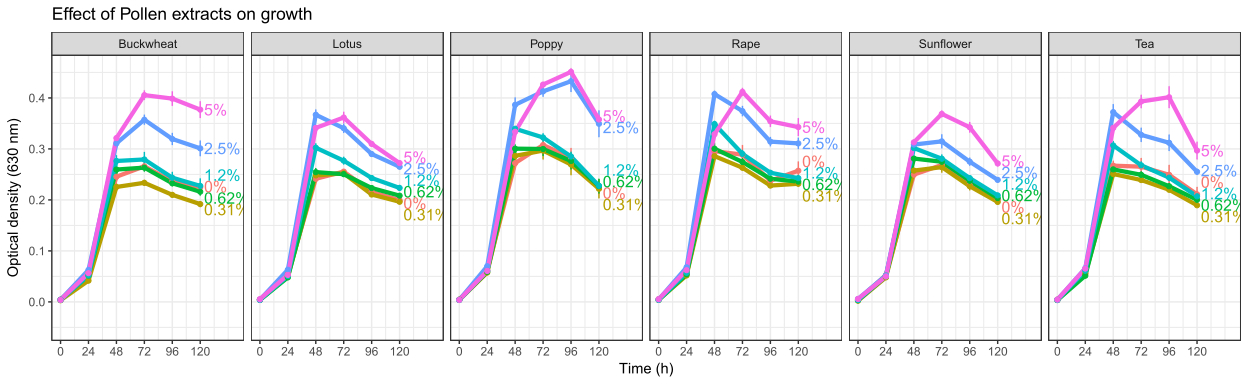


**Supplementary Figure 1.** Growth curves for the individual pollen experiment.

The x-axis shows time of the experiment. Points and error bars show means and standard errors for n=8 replicates per treatment concentration. Readings were taken at 24 h intervals. Lines represent different concentrations.


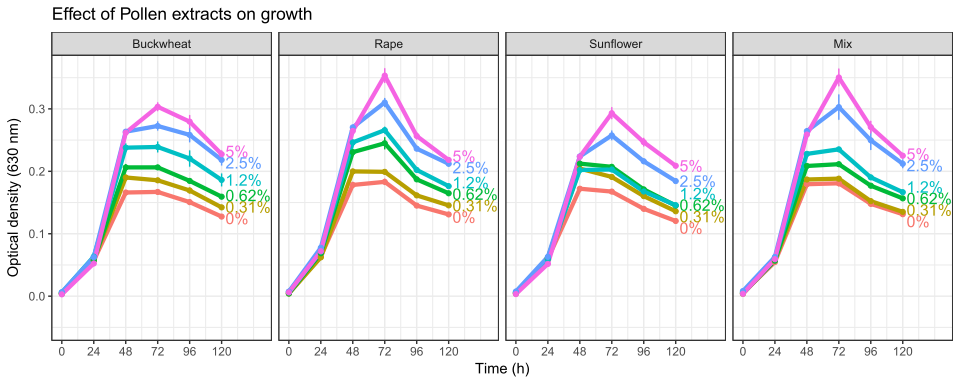


**Supplementary Figure 2.** Growth curves for the mixed-pollen experiment

The x-axis shows time of the experiment. The "mix" treatment consisted of equal proportions of buckwheat, rape, and sunflower pollen extracts. Points and error bars show means and standard errors for n=8 replicates per treatment concentration. Readings were taken at 24 h intervals. Lines represent different concentrations.


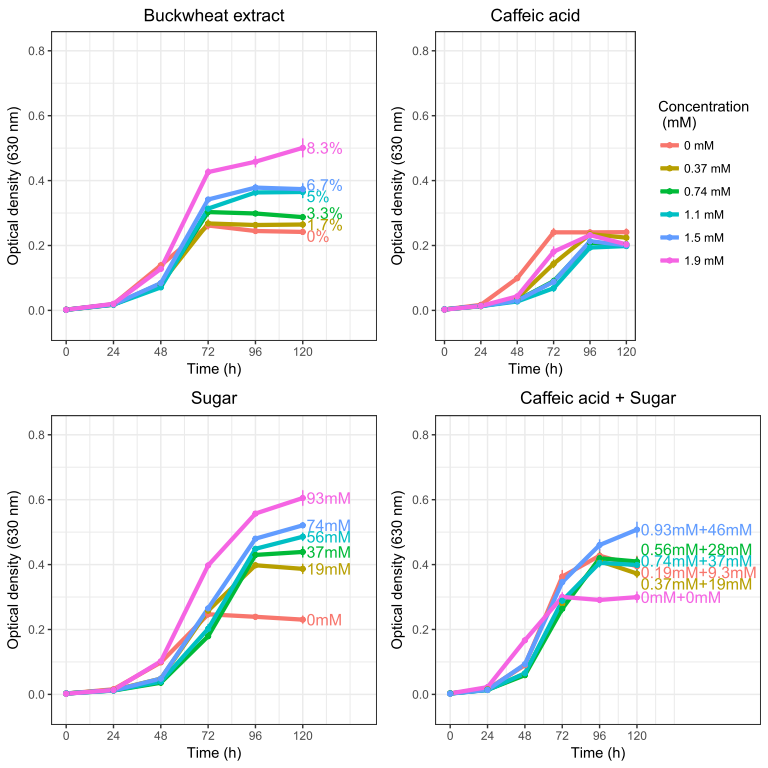


**Supplementary figure 3.** Growth curves for the chemical additions experiment.

The x-axis shows time of the experiment. Both sugar and caffeic acid were dissolved in 50% methanol. The "sugar" treatment consisted of equimolar amounts of glucose and fructose. Points and error bars show means and standard errors for n=5 replicates per treatment concentration. Readings were taken at 24 h intervals. Lines represent different concentrations.


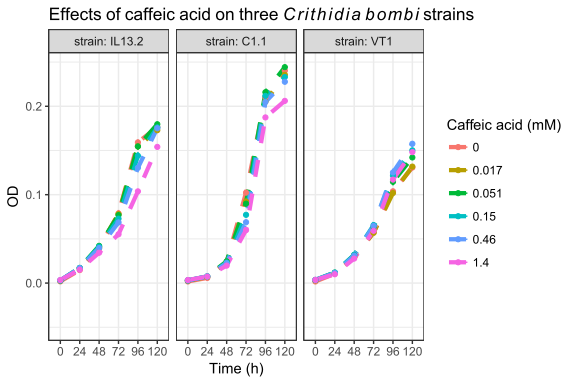


**Supplementary figure 4.** Effects of caffeic acid (aqueous) on three strains of *Crithidia bombi*.

Strain IL13.2 is the cell line used in the preceding experiments reported in the manuscript. Although some inhibition was realized at the highest concentration (1.4 mM), none of the tested concentrations resulted in >50% inhibition. Points and error bars show means and standard errors for n=5 replicates per treatment concentration. Readings were taken at 24 h intervals. Lines represent different concentrations.
